# Supplementary material for: Folding and unfolding: A topological framework for understanding intangible cultural heritage tourism in urban villages - The case of chebei dragon boat scenery, Guangzhou, China
Source: PLoS One. 2026 Jan 20;21(1):e0339564. doi: 10.1371/journal.pone.0339564 (PMC12818644; doi:10.1371/journal.pone.0339564)
Supplement: S1 File — (DOCX) [file pone.0339564.s001.docx]

**S1: Comprehensive Interview Guide**

**Informed Consent Statement**

**Research Overview** This interview is part of a research study examining how traditional cultural practices in urban villages adapt to tourism development. The research focuses on dragon boat culture in Chebei Village, Guangzhou, and is conducted by XXXX from XXXX for academic purposes.

**Your Participation** Your participation is completely voluntary. This interview will take 45-120 minutes and will be audio recorded with your permission. You may decline to answer any question or end the interview at any time without consequences.

**Privacy Protection** Your identity will be protected through anonymization. You will be assigned a code number, and your real name will never appear in any publications. Direct quotes may be used but will only be attributed to general categories such as "community resident" or "business owner."

**Data Use** The information you provide will be used only for academic research, including potential publication in scholarly journals. Audio recordings will be stored securely and deleted after five years. A summary of research findings will be shared with the Chebei Village community.

**Your Rights** You have the right to review any quotes attributed to you before publication. You may withdraw from the study at any time and request that your data not be used. For questions or concerns, you may contact XXXX.

**Consent Confirmation** Do you understand this information and agree to participate in this interview? Do you consent to audio recording?

**Section 1: Background and Cultural Connection (All Participants)**

1.您与车陂村的关系是什么？住了多长时间？(What is your relationship to Chebei Village? How long have you lived here?)

2.您是如何接触到龙舟文化的？(How did you first encounter dragon boat culture?)

3.您在龙舟活动中扮演什么角色？(What role do you play in dragon boat activities?)

4.对您来说，龙舟文化意味着什么？(What does dragon boat culture mean to you personally?)

**Section 2: Cultural Understanding and Transmission (Local Residents, Inheritors, Association Members)**

5. 您能描述一下车陂龙舟的传统仪式和意义吗？(Can you describe the traditional rituals and meanings of Chebei dragon boats?)

6. 龙舟文化是如何传承给年轻一代的？(How is dragon boat culture transmitted to younger generations?)

7. 您觉得车陂龙舟文化最独特的地方是什么？(What do you think is most unique about Chebei dragon boat culture?)

8. 传统的龙舟仪式在今天还保持着吗？有什么变化？(Are traditional dragon boat rituals still maintained today? What changes have occurred?)

**Section 3: Change and Continuity (Long-term Residents)**

9. 这些年来，龙舟景活动发生了哪些变化？(What changes have occurred in dragon boat activities over the years?)

10. 哪些传统元素保持不变？(Which traditional elements have remained unchanged?)

11. 城市化对龙舟传统产生了什么影响？(What impact has urbanization had on dragon boat traditions?)

12. 您如何看待传统与现代的结合？(How do you view the combination of tradition and modernity?)

**Section 4: Spatial and Environmental Dimensions (Residents, Officials)**

13. 车陂涌的环境变化对龙舟活动有什么影响？(How have environmental changes to Chebei Creek affected dragon boat activities?)

14. 村里的物理空间是如何支持龙舟活动的？(How do the village's physical spaces support dragon boat activities?)

15. 您如何看待村庄空间的变化？(How do you view changes in village space?)

16. 哪些空间对龙舟文化最重要？(Which spaces are most important for dragon boat culture?)

**Section 5: Economic Dimensions (Business Owners, Officials, Association Leaders)**

17. 龙舟活动需要什么资源支持？(What resources are needed to support dragon boat activities?)

18. 活动是如何筹资和组织的？(How are activities funded and organized?)

19. 龙舟景对社区经济有什么影响？(What economic impact does the dragon boat festival have on the community?)

20. 您如何看待龙舟文化的经济价值？(How do you view the economic value of dragon boat culture?)

**Section 6: Social and Community Dynamics (All Participants)**

21. 不同群体（本地人、租客、商家）如何参与龙舟活动？(How do different groups - locals, tenants, businesses - participate in dragon boat activities?)

22. 传统的家族和宗祠结构在今天发挥什么作用？(What role do traditional family and ancestral hall structures play today?)

23. 社区决策是如何做出的？(How are community decisions made?)

24. 外来人口如何融入龙舟文化？(How do migrants integrate into dragon boat culture?)

**Section 7: Tourism Development (Tourists, Business Owners, Cultural Workers)**

25. 您如何看待旅游对龙舟文化的作用？(How do you view tourism's role in dragon boat culture?)

26. 旅游给传统带来了什么机遇和挑战？(What opportunities and challenges does tourism bring to traditions?)

27. 游客和居民体验文化的方式有什么不同？(How do tourists and residents experience culture differently?)

28. 您认为应该如何平衡文化保护和旅游发展？(How do you think cultural protection and tourism development should be balanced?)

**Section 8: Cultural Experience and Meaning (All Participants)**

29. 参与龙舟活动对您个人意味着什么？(What does participating in dragon boat activities mean to you personally?)

30. 您如何向外人解释这个传统的意义？(How do you explain the significance of this tradition to outsiders?)

31. 龙舟景期间的氛围和感受是怎样的？(What is the atmosphere and feeling like during the dragon boat festival?)

32. 什么让您感到这个传统的延续？(What makes you feel the continuity of this tradition?)

**Section 9: Future Vision and Sustainability (All Participants)**

33. 您如何看待龙舟传统的未来？(How do you view the future of dragon boat traditions?)

34. 什么应该保持不变，什么可以改变？(What should remain unchanged, and what can change?)

35. 旅游应该在文化保护中发挥什么作用？(What role should tourism play in cultural protection?)

36. 您对下一代有什么希望和担忧？(What hopes and concerns do you have for the next generation?)
