# Supplementary material for: Folding and unfolding: A topological framework for understanding intangible cultural heritage tourism in urban villages - The case of chebei dragon boat scenery, Guangzhou, China
Source: PLoS One. 2026 Jan 20;21(1):e0339564. doi: 10.1371/journal.pone.0339564 (PMC12818644; doi:10.1371/journal.pone.0339564)
Supplement: S2 File — (DOCX) [file pone.0339564.s002.docx]

**S2: Detailed Grounded Theory Coding Structure**

**Open Coding Categories (127 Initial Concepts)**

**Cultural Concepts (文化类概念):**

- 龙舟情结 (Dragon boat complex) - Deep emotional identification and cultural belonging
- 河涌污染记忆 (Creek pollution memory) - Collective memory of environmental degradation and restoration
- 仪式压缩 (Ritual compression) - Condensation of traditional ceremonies in modern contexts
- 水路关系 (Water road relationships) - Spatial expression of inter-village social networks
- 象征密度 (Symbolic density) - Multiple layers of meaning in cultural symbols
- 祖先桨连接 (Ancestral paddle connection) - Temporal bridging through inherited objects
- 集体鼓声体验 (Collective drumming experience) - Synchronized ritual participation
- 龙舟装饰象征 (Dragon boat decoration symbolism) - Cultural identity markers

**Social Concepts (社会类概念):**

- 外来融入 (Migrant accommodation) - Cultural adaptation strategies for newcomers
- 代际传承 (Intergenerational transmission) - Cultural knowledge transfer across generations
- 宗族经济实力 (Clan economic strength) - Traditional resource mobilization capacity
- 节庆聚会义务 (Festival hospitality obligations) - Traditional reciprocity systems
- 包容性动态 (Inclusive dynamics) - Expansion of cultural participation beyond bloodlines
- 青年技能传递 (Youth skill transmission) - Training and knowledge transfer to younger generations

**Spatial Concepts (空间类概念):**

- 游客拍照干扰 (Tourist photography interference) - Visitor behavior and cultural boundaries
- 分布式文化基础设施 (Distributed cultural infrastructure) - Spatial dispersion of cultural elements
- 祠堂文化中心化 (Ancestral hall centralization) - Traditional authority spaces
- 观龙台建设 (Dragon watching platform construction) - Tourism infrastructure development
- 水质修复影响 (Water quality restoration impact) - Environmental change and cultural practice

**Economic Concepts (经济类概念):**

- 文化展览创新 (Cultural exhibition innovation) - Heritage interpretation methods
- 龙舟标语赞助 (Dragon boat banner sponsorship) - Commercialization and tradition integration
- 租金依赖模式 (Rental dependency model) - Village collective economic structure
- 旅游乘数效应 (Tourism multiplier effects) - Economic linkages around heritage
- 文创产品开发 (Cultural creative product development) - Commercial cultural adaptation

**Axial Coding: Six Main Categories with Detailed Subcategories**

**1. ICH Evolution (非遗演化)**

- Historical Imprints (历史印记): Transformation from ritual to symbol
  - Cosmological foundations in water deity worship
  - Social consolidation through inter-village networks
  - Cultural symbolization creating meaning systems
- Ritual Refinement (仪式提炼): Continuous adaptation of practices
  - Ceremony compression maintaining essential meanings
  - Seasonal alignment despite urban livelihoods
  - Sacred-secular integration in contemporary contexts
- Symbolic Density (象征密度): Multiple meanings in cultural forms
  - Visual identity markers in boat decorations
  - Hierarchical expressions through racing formations
  - Moral value embodiment in ritual protocols

**2. ICH Presentation (非遗呈现)**

- Physical Environment (物质环境): Material conditions for practice
  - Creek dimensions enabling large-scale events
  - Environmental restoration supporting cultural revival
  - Infrastructure enhancement respecting traditional requirements
- Economic Environment (经济环境): Resource base and funding
  - Collective property providing financial foundation
  - Festival cost requirements and funding mechanisms
  - Economic pressures and adaptation strategies
- Social Environment (文化环境): Networks and relationships
  - Demographic diversity in cultural participation
  - Migrant integration and cultural inclusion
  - Youth cultural fusion across origin differences

**3. ICH Experience (非遗体验)**

- Embodied Knowledge (身体化知识): Skills through practice
  - Physical learning transcending linguistic barriers
  - Muscle memory preserving cultural techniques
  - Tourist experiential engagement possibilities
- Collective Effervescence (集体欢腾): Shared emotional states
  - Synchronized action generating group consciousness
  - Cross-cultural experiential intensity
  - "Dragon energy" as cultural constant
- Temporal Connection (时间连接): Links across generations
  - Artifact handling creating ancestral presence
  - Movement repetition maintaining historical continuity
  - "Thick time" experiential collapse

**4. Cultural Space Reproduction (文化空间再生产)**

- Narrative Innovation (叙事创新): New storytelling forms
  - Video media capturing experiential essence
  - Social media engagement expanding cultural reach
  - Contemporary aesthetics maintaining traditional themes
- Spatial Reinterpretation (空间重新阐释): Evolving space meanings
  - Museum design translating tacit knowledge
  - Exhibition flow following cultural rhythms
  - Interactive displays enabling visitor participation
- Experience Design (体验设计): Participatory opportunities
  - Simulator technology enabling skill learning
  - Workshop programming teaching traditional crafts
  - Festival preparation involving tourist participation

**5. Power Space Reproduction (权力空间再生产)**

- Hybrid Governance (混合治理): Traditional-modern authority
  - Legal registration maintaining cultural leadership
  - Bureaucratic compliance preserving community trust
  - Cultural brokerage between regulatory and traditional demands
- Inclusive Participation (包容性参与): Beyond clan boundaries
  - Membership expansion through demonstrated commitment
  - Merit-based inclusion replacing bloodline inheritance
  - Constitutional reform accommodating demographic change
- Multi-scalar Networks (多尺度网络): Local-global connections
  - International federation membership providing resources
  - Technical standards adoption maintaining local characteristics
  - Global platform assertion of distinctive identity

**6. Capital Space Reproduction (资本空间再生产)**

- Asset Transformation (资产转换): Cultural use conversion
  - Agricultural land hosting cultural facilities
  - Traditional buildings becoming tourism infrastructure
  - Community ownership maintaining cultural control
- Value Chain Development (价值链发展): Economic linkages
  - Primary attraction generating secondary spending
  - Local business benefit through tourism multipliers
  - Distribution conflicts requiring governance intervention
- Symbolic Capital Conversion (象征资本转换): Prestige to economic value
  - ICH certification creating marketing advantages
  - Cultural reputation enhancing property values
  - Official recognition generating government investment

**Selective Coding: Theoretical Integration**

- **Core Category**: Cultural Experience as Topological Invariant
- **Central Phenomenon**: Spatial reproduction through community-controlled tourism development
- **Causal Conditions**: Urbanization pressures threatening cultural continuity + Tourism opportunities for cultural sustainability
- **Context**: Urban village transformation within broader heritage tourism development trends
- **Intervening Conditions**: Government policies + Community organizational capacity + Market demand for authentic cultural experiences
- **Action/Interaction Strategies**: Culture-power-capital matrix dynamics enabling adaptive cultural reproduction
- **Consequences**: Sustainable cultural continuity + Economic diversification + Community empowerment through cultural tourism

**Theoretical Relationship Mapping:** The coding structure reveals how topological folding (structural elements) enables unfolding (reproduction processes) through maintenance of experiential invariants while adapting material and organizational forms to contemporary contexts. The "2-6-18" logic demonstrates systematic relationships among individual cultural practices, community-level governance structures, and regional development patterns that can be theoretically generalized while remaining sensitive to local cultural specificity.
