# Supplementary material for: Folding and unfolding: A topological framework for understanding intangible cultural heritage tourism in urban villages - The case of chebei dragon boat scenery, Guangzhou, China
Source: PLoS One. 2026 Jan 20;21(1):e0339564. doi: 10.1371/journal.pone.0339564 (PMC12818644; doi:10.1371/journal.pone.0339564)
Supplement: Attachment — Submitted filename: Response to Reviewers20251102.docx [file pone.0339564.s004.docx]

**Response to Reviewers**

**Dear Dr. You, Academic Editor, and Esteemed Reviewers,**

Thank you for your meticulous review and the invaluable, constructive feedback on our manuscript. We have benefited greatly from your insights. In accordance with your recommendations, we have undertaken a careful and rigorous revision of the paper, as detailed in the uploaded file "Revised Manuscript with Track Changes."

Below, we detail our point-by-point responses to the comments from the journal office and all reviewers, referencing the specific changes made in the revised manuscript.

**Part 1: Response to Journal Requirements**

**1. Journal Requirement (Style):**

*Please ensure your manuscript meets PLOS ONE's style requirements...*

**Response:** We have thoroughly reviewed the PLOS ONE style templates and revised the manuscript, including file naming, formatting, and section structure, to ensure full compliance with the journal's style requirements.

**2. Journal Requirement (Ethics Statement):**

*In the Methods' ethical statement, you have indicated that verbal consent was obtained. Please provide more details on how this consent was documented and witnessed, and state if this was approved by the IRB.*

**Response:** We thank the journal office for this important query. We have significantly strengthened our Ethics Statement. As shown in the "Research Quality and Ethical Considerations" section of our revised manuscript, we have replaced the previous statement with a more detailed one, which is now supported by an official **Ethical Review Exemption Certificate** (Ref: 20251028) issued by the Academic Committee of Guangdong University of Education (our institution's review board). This certificate confirms the study was classified as "minimal risk research" and explicitly approved the research plan, including the verbal consent procedure.

The revised Ethics Statement in the manuscript now reads: *"This study was reviewed and approved by the Academic Committee of Guangdong University of Education (Ethical Review Exemption Certificate, Ref: 20251028). The committee determined that the research, which involved interviews and participant observation with non-vulnerable adult populations, constituted 'minimal risk research' and was exempt from routine supervision. All participants were adults. Prior to interviews, the research purpose, methodology, data usage, privacy protection measures, and participant rights (including unconditional withdrawal) were fully disclosed. Verbal consent was obtained from each participant, as this method was reviewed and approved by the ethics committee for this low-risk study. All interview data have been anonymized to protect participant privacy and ensure confidentiality."*

We will also upload a translation of this ethics certificate as a supporting file.

**3. Journal Requirement (Code Sharing):**

*Please note that PLOS One has specific guidelines for sharing code...*

**Response:** We confirm that our study did not generate any custom code or software. The analysis was conducted using standard qualitative data analysis software (NVivo 12). Therefore, this requirement is not applicable to our manuscript.

**4. Journal Requirement (Figure 2 Copyright):**

We note that Figure 2 in your submission contains map/satellite images that may be copyrighted... We cannot publish previously copyrighted maps or satellite imagery created using proprietary data (e.g. Google software...)

**Response:** We thank the journal office for this critical reminder. To ensure full compliance with the CC BY 4.0 license, we have **completely revised the map figure** (now **Figure 3** in the revised manuscript). As stated in the new figure caption, the map was redrafted using QGIS software, and all map data is sourced from **Natural Earth (public domain)**. This new figure replaces the previous one and resolves any copyright concerns.

**Part 2: Response to Reviewer #1**

**1.1. Reviewer Comment (Theory Applicability & Limitations):**

*(1) ICH inheritance involves multiple dimensions... Although the topological "folding-unfolding" model can explain the transformation of cultural meanings, it is difficult to fully cover specific issues... such as institutional contradictions... and economic exploitation... the topological framework has relatively weak analysis of "how capital distorts cultural meanings".*

**Response:** We fully agree with this insightful critique. The topological model's strength is in revealing internal structural logic, not in providing a deep causal analysis of external political-economic forces. To address this, we have made two key revisions in the manuscript:

- **First,** in **Section 1.2 ("Spatial Production Theory and the Culture-Power-Capital Matrix")**, we have added a new paragraph explicitly stating the model's limitations. We clarify that for deeper analysis of institutional contradictions or the distorting effects of capital, "a more specialized theoretical toolkit, such as institutional analysis or political economy, would be required."
- **Second,** in **Section 4 ("Discussion")**, we have added a new critical analysis that directly addresses the potential negative impacts of capital, including the risks of **"cultural flattening and hollowing out,"** the **"weakening of community agency,"** and the **"degenerating from a folk ritual... into a pure tourism attractor."**

**1.2. Reviewer Comment ("Invariant" as Metaphor / Lack of Evidence):**

*(2) "Invariants" in topology... have strict mathematical definitions, while the paper defines "cultural experience" as a "topological invariant", which is essentially a metaphorical borrowing... "cultural experience" is highly subjective... Therefore, further empirical evidence is needed...*

**Response:** We thank the reviewer for this crucial point. We have revised the manuscript to clarify our methodological position and strengthen our evidence.

- **Clarifying the Metaphor:** In **Section 1.2 ("Theoretical Bridging")**, we now explicitly state that our use of "topological invariant" is a **"metaphorical borrowing"** and not a strict mathematical equivalence. We redefine it in this socio-cultural context as "the core element that maintains a stable, connective function throughout dynamic transformation—which this paper identifies as 'cultural experience.'"
- **Strengthening Evidence:** In **Section 3.1 ("ICH Experience")**, we have added a new concluding paragraph to *justify why* "cultural experience" acts as this invariant. We use interview data to contrast the perspectives of **local residents (1d-9)**, **migrant tenants (1d-6)**, and **tourists (1i-1, 1i-2)**. This evidence demonstrates that despite different subjective viewpoints, a *shared, stable* core experience of collective emotion and cultural attraction exists, which functions as the stable "connection point" for the community.

**1.3. Reviewer Comment (Methods: Participant Observation):**

*...the feelings obtained from participant observation must have influenced the construction of coding categories... which needs to be explained...*

**Response:** We fully agree. The role of participant observation was understated. In **Section 2 ("Participant Observation")**, we have added a new paragraph explaining that this immersive fieldwork (e.g., joining training, rituals, and communal meals) provided a **"sensory basis"** and **"contextual clues"** for the coding process, which was essential for capturing tacit cultural logic and informing the development of our coding categories.

**1.4. Reviewer Comment (Methods: Interview/Coding Link):**

*The relationship between the questions listed in the paper's interview outline and the coding categories is not clearly explained... it is difficult to derive such rich coding categories from the currently presented interview outline.*

**Response:** This is an excellent point regarding methodological transparency. To address this, we have added a **new table (now Table 1)** in **Section 2 ("Data Analysis Procedures and Systematic Coding")** titled "**Example of Correspondence Between Core Interview Questions and Coding Categories.**" This table visually demonstrates how the core interview questions directly correspond to and generate the rich coding categories presented in the study, making the "method-data-analysis" chain clear and robust. (Please note: This addition required renumbering all subsequent tables throughout the manuscript).

**Part 3: Response to Reviewer #2**

**2.1. Reviewer Comment (Engagement with ICH Literature):**

*...the manuscript... falls short of fully engaging with international debates and scholarly literature in the field—particularly regarding concepts such as living heritage, community participation, and cultural sustainability... A clearer conceptual framework is needed...*

**Response:** We sincerely thank the reviewer for this feedback. We acknowledge that our engagement with international ICH literature was insufficient. We have made significant additions to **Section 1.2 ("Theoretical Bridging")** to address this. We now explicitly discuss the core international concepts of **"living heritage"** and **"community participation,"** referencing **UNESCO's 2003 Convention** and related international scholarship (e.g., [23-25]). This revision situates our study firmly within this global academic dialogue.

**2.2. Reviewer Comment (Theoretical Framing & Literature Foundation):**

*The authors are proposing a... “culture–power–capital” matrix. However, the conceptual framework requires stronger support from relevant English-language literature... Terms like cultural space and symbolic capital have rich scholarly lineages...*

**Response:** We fully agree. To systematically strengthen our theoretical foundation, we have made significant additions to **Section 1.2 ("Spatial Production Theory and the Culture-Power-Capital Matrix")**. We now clearly articulate that the "culture-power-capital" matrix is rooted in established theoretical lineages, drawing from **Lefebvre [29]** (spatial production), **Foucault [32]** (power/space), and **Bourdieu [30, 31]** (field/capital). We have added these key citations to connect our framework to the broader academic conversation.

**2.3. Reviewer Comment (Research Participants / Sampling):**

*...migrants constitute over 80% of Chebei’s current population... However, the study includes only six migrant participants... This sampling limits the analysis... A more balanced and inclusive sampling strategy would better reveal the contested, negotiated, and pluralistic nature of ICH...*

**Response:** The reviewer correctly points out this limitation. While we cannot retroactively change the sample, we have revised the manuscript to better leverage the existing data and make our sample structure transparent:

- **First,** in **Section 2 ("In-depth Interviews")**, we have added a new paragraph detailing the participant composition and explicitly categorizing them into "**Traditional local stakeholders**" (N=21, 55.3%) and "**External and associated populations**" (N=17, 44.7%).
- **Second,** in our analysis (e.g., **Section 3.1, "ICH Experience"**), we now more intentionally highlight and contrast the views of **migrant tenants (1d-6)** and **tourists** with those of traditional stakeholders to demonstrate the *negotiated* and *pluralistic* nature of the ICH space.

**2.4. Reviewer Comment (Critical Dialogue & Tourism Risks):**

*The current analysis tends to emphasize the positive role of tourism... without addressing potential risks such as cultural appropriation, or loss of community agency... A more nuanced, dialectical perspective would strengthen the critical depth...*

**Response:** We fully agree that our original analysis was overly optimistic. As noted in our response to R1.1, we have made a major revision to **Section 4 ("Discussion")**. We have added a new, substantial paragraph that critically analyzes the "urban renewal + cultural tourism" model, drawing from international research. We now explicitly identify and discuss potential risks, including the **"flattening of cultural symbols,"** the **"weakening of community agency,"** and the **"erosion by commercial interests."** This adds the necessary critical depth and dialectical perspective that the reviewer rightly called for.

**Part 4: Response to Reviewer #3**

**4.1. Reviewer Comment (Dynamic Heritage Premise):**

*...the idea... that “Traditional approaches often conceptualize heritage spaces as static entities”... is somewhat flawed... the idea that there can be continuity within change seems to me to be generally accepted in social sciences... and should rather be a starting point.*

**Response:** We fully agree with and thank the reviewer for this fundamental theoretical correction. The reviewer is entirely correct that "continuity within change" is the consensus in modern heritage studies, and our "static vs. dynamic" premise was flawed. We have made two systemic revisions to fix this:

- **First,** in **Section 1.2 ("Theoretical Bridging")**, we have rewritten the introduction to *start from* the accepted consensus that ICH is dynamic (**"living heritage"**), citing the UNESCO convention (as also noted by R2.1).
- **Second,** we have **deleted the flawed sentence** (original P7, lines 133-136) that set up this false dichotomy. We now position our topological framework not as a new *idea*, but as a novel *analytical language* and *heuristic metaphor* to better describe *how* this accepted dynamic process operates.

**4.2. Reviewer Comment (Topological Model as Metaphor / Lack of Dialogue):**

*...the article does not appropriately acknowledge the extensive body of literature in social sciences that mobilize the reference to topology... (Lury et al., Martin and Secor... Phillips)... it is not clear is if the authors use the reference to topology as... analogy, or simply as a metaphor... what the article also needs to make very clear... is that the approach they propose applies to... the context of the management of ICH.*

**Response:** We sincerely thank the reviewer for these insightful recommendations, which have significantly sharpened the manuscript's focus. We have made the following key revisions based on your suggestions:

- **Engaging with "Topological Turn":** In **Section 1.2 ("Theoretical Bridging")**, we now explicitly situate our research within the "topological turn" in social sciences, referencing the scholars you suggested (e.g., **Lury et al. [24], Martin & Secor [23], Phillips [25]**) to connect our work to this broader academic dialogue.
- **Clarifying "Metaphor vs. Tool":** In **Section 1.2**, we now explicitly state that we are using topology primarily as a **"heuristic metaphor"** and **"analytical language"** [23-25] to describe structure and relations, not as a strict mathematical tool. This directly addresses the reviewer's ambiguity.
- **Focusing on "ICH Management":** We fully agree that the framework's primary value is in management. We have sharpened this focus throughout the manuscript. The **Conclusion (Section 5)** now opens by stating the research offers "a systematic approach for **heritage tourism management and policy development**," and the **Policy Implications (Section 4)** section is structured around this practical application. This justifies the need for a model that can identify a stable "invariant" (cultural experience) within a process of managed change.

We believe that these comprehensive revisions, as detailed in the "Revised Manuscript with Track Changes" file, have addressed all the reviewers' concerns. We have significantly strengthened the manuscript's theoretical grounding, methodological transparency, and critical depth. We thank you once again for your invaluable guidance.

Sincerely,

Shengchao Li Corresponding Author Guangdong University of Education
