# Supplementary material for: Folding and unfolding: A topological framework for understanding intangible cultural heritage tourism in urban villages - The case of chebei dragon boat scenery, Guangzhou, China
Source: PLoS One. 2026 Jan 20;21(1):e0339564. doi: 10.1371/journal.pone.0339564 (PMC12818644; doi:10.1371/journal.pone.0339564)
Supplement: Attachment — Submitted filename: Response to Reviewers20251125.docx [file pone.0339564.s005.docx]

Dear Dr. You (Academic Editor),

We seek to express our sincere gratitude for the opportunity to revise our manuscript one final time. We have made every effort to address the specific concern raised by Reviewer #2 regarding the limitation and consequence of our data collection.

Below is our detailed response and the specific changes made to the manuscript.

**Response to Reviewers**

**Reviewer #2 Comment:** "The authors need to explain clearly the limitation and consequence of the data collection in this research that although migrants constitute 80% of the resident of Chebei, only 6 migrant tenants and 3 tourists have been studied."

**Response:** We fully accept this critical observation. We agree that the discrepancy between the demographic reality (80% migrants) and our qualitative sample size (6 migrants, 3 tourists) creates a specific limitation that must be explicitly acknowledged and analyzed. In the revised "Limitations" section (Lines 1194-1207), we have added a detailed explanation of this issue. We clarified that:

1.	The Limitation: The sample is skewed towards local stakeholders because they remain the primary gatekeepers and active practitioners of the ritual.

2.	The Consequence: The findings may over-emphasize the "insider" perspective of cultural inheritance, potentially underestimating the challenges or specific marginalization experienced by the migrant majority. We have explicitly stated that this limits the generalizability of our findings regarding the "inclusive" nature of the space and calls for future quantitative validation.

**Added Text in Manuscript:** Furthermore, regarding the sampling structure, we acknowledge a disparity between the demographic composition of the village and our respondent pool. Although migrants constitute approximately 80% of Chebei's resident population, our qualitative sample included only 6 migrant tenants and 3 tourists. This limitation stems from the fact that the core rituals and organizational activities of the Dragon Boat Festival remain dominated by local clans with ancestral ties. The consequence of this sampling limitation is that our analysis may disproportionately reflect the perspectives of active cultural insiders and traditional stakeholders, potentially underrepresenting the nuanced, lived experiences of the silent majority—the migrants. While our findings suggest that "cultural experience " acts as a bridge, the specific barriers or passive forms of participation experienced by this large demographic may not be fully captured. Future studies should employ broader quantitative surveys or targeted sampling of non-local residents to rigorously assess the inclusivity of such ICH spaces.

We believe this assessment of our study's boundaries significantly enhances the rigor of the paper.

Sincerely, Shengchao Li
